# Postoperative urinary tract infections and the role of Tc-99 m-MAG3 renography scans following construction of a urinary diversion

**DOI:** 10.1007/s11255-026-05049-9

**Published:** 2026-03-17

**Authors:** Julie Sofie Riemann, Ulla Nordström Joensen, Andreas Røder, Maja Vejlgaard

**Affiliations:** 1 Urological Research Unit, Department of Urology, Centre for Cancer and Organ Diseases, Copenhagen University Hospital – Rigshospitalet, Copenhagen, Denmark; 2 Department of Clinical Medicine, Faculty of Health and Medical Sciences, University of Copenhagen, Copenhagen, Denmark

**Keywords:** Postoperative complications, Renography, Tc-99 m-MAG3, Urinary diversion, Urinary tract infection

## Abstract

**Purpose:**

To identify risk factors for postoperative urinary tract infection (UTI) within 90 days following construction of a urinary diversion, and to assess the association between UTIs and results of renography scans 6–8 weeks postoperatively.

**Methods:**

A retrospective review of 612 patients who received a urinary diversion with or without radical cystectomy at Rigshospitalet, Denmark between January 2019 and January 2025. The primary outcome was UTI within 90 days after surgery, defined as presence of relevant symptoms, urine culture ≥ 10,000 CFU, and treatment with antimicrobials. Delayed excretion on Tc-99 m-MAG3 renography was defined as documentation thereof in the medical records by a urologist. Cox regression and multivariable logistic regression were performed to identify risk factors.

**Results:**

A total of 346 UTIs occurred in 242 patients (40%) within 90 days after surgery. Bacteremia was present in 111 (32%) of the UTIs. Cox regression showed that female sex (HR = 1.5), BMI 25–30 kg/m^2^ (HR = 1.6), preoperative diabetes (HR = 1.7), orthotopic neobladder (HR = 2.1) or continent cutaneous reservoir (HR = 3.4), and early stent dislodgement (HR = 2.0) were all associated with an increased risk of UTI. On multivariable logistic regression, delayed excretion on postoperative renography was associated with higher UTI rate (OR = 1.7), whereas asymmetrical functional distribution was not (OR = 0.96).

**Conclusion:**

Postoperative UTI after urinary diversion is common and often complicated by bacteremia. Several risk factors for UTI within 90 days were identified, with the highest HR for continent diversions. Delayed excretion on renography was associated with occurrence of UTI, indicating that UTI is related to postoperative urine obstruction.

**Supplementary Information:**

The online version contains supplementary material available at 10.1007/s11255-026-05049-9.

## Introduction

Construction of a urinary diversion is one of the most complex surgical procedures routinely performed in the urological field. Postoperative complications, especially infections, are common. Urinary diversions are indicated for patients who undergo cystectomy or who have a permanently non-functional bladder. Bladder cancer stands as the predominant indication for construction of a urinary diversion, but the procedure can also be used to treat other bladder-invading malignant tumors, such as prostatic, cervical, or colorectal cancers, as well as benign conditions, including adverse effects of radiotherapy, neurogenic bladder dysfunction, or painful bladder syndrome.

Different types of urinary diversions exist. The most used in Denmark is the ileal conduit, whereas continent reservoirs, such as orthotopic neobladder and continent cutaneous reservoir, are performed in under 5% of bladder cancer patients [1]. A Mitrofanoff procedure is used in some cases where all or most of the bladder can be salvaged, and ureterocutaneostomies are used in cases where use of a bowel segment for the diversion is not possible.

Around 60% of patients experience any complication within 90 days, most commonly infectious [2, 3]. Several factors make this patient group particularly susceptible to urinary tract infections (UTIs), including the use of intestinal substitution for the reconstruction, the increased exposure to the surroundings when a urostomy is used, risk of urinary stasis, or frequent catheterization when a continent diversion is used. Risk factors for developing UTI after construction of a urinary diversion have previously been reported, but results are conflicting, and clear risk factors are not internationally established [4–7].

At our site, follow-up of patients has historically included a Technetium-99 m mercaptoacetyltriglycine (Tc-99 m-MAG3) renogram (renography scan) 6–8 weeks postoperatively and subsequently annually until no longer indicated. The rationale for performing postoperative renography scans was to evaluate the urine drainage through the reconstructed urinary tract to allow for timely intervention, usually involving stenting of the ureter(s) to minimize permanent damage to kidney function. Furthermore, it has been the belief that postoperative UTIs in patients with a urinary diversion are linked to a mechanical obstruction in the urinary tract for which renography scans are useful to identify high-risk patients [8, 9]. In recent years, the clinical value of postoperative renography scans has been questioned, but little data exist on whether renography scans contribute meaningfully to the postoperative follow-up course.

Our group previously published a paper on microbial trends at infection-related readmissions following cystectomy in bladder cancer patients [10]. We here present a subsequent contemporary cohort including non-bladder cancer and non-cystectomy patients with additional data on the postoperative course of this cohort. The study aims to assess risk factors for developing UTI following surgical construction of urinary diversion and to assess the relation between UTIs and results of postoperative renography scans. We hypothesize that patients with continent diversion types are at a higher risk of postoperative UTI and that abnormal renography scans are related to UTIs.

## Materials and methods

This study is a retrospective observational study. All patients who underwent construction of a urinary diversion at Rigshospitalet in Copenhagen, Denmark between January 2019 and January 2025 were identified through central extraction of Civil Registration Numbers. The cohort includes both robot-assisted and open surgery, all indications for undergoing the procedure, both benign and malignant, cystectomy and non-cystectomy patients, and all types of urinary diversion procedures, ileal conduit, orthotopic neobladder, cutaneous continent reservoir, ureterocutaneosstomy and Mitrofanoff procedure. Patients under the age of 18 are excluded from the cohort. Detailed descriptions of all types of diversions are included in Supplementary material 1.

Baseline data were extracted from the Danish Electronic Medical Records (EMR), including information on sex, age, body mass index, smoking status, Charlson Comorbidity Index (CCI) without age-adjustment, ASA-score, preoperative comorbidities (diabetes and hypertension), and preoperative renal function measured in eGFR (using the CKD-EPI equation, 2021), and categorized into Chronic Kidney Disease (CKD) stage according to the K/DOQI guidelines, urinary diversion type, indication for surgery, preoperative chemotherapy (neoadjuvant or downstaging), history of radiotherapy to the pelvis, surgical technique, and, if applicable, cancer stage [11].

The standard-of-care follow-up program is illustrated in Supplementary material 2 [12]. The standard program was subject to adjustment according to patient compliance and/or in the case of a single-functioning kidney. Planned removal of stents was generally 10 days postoperatively for conduits and 3 weeks postoperatively for continent diversions. Any early removal or spontaneous dislodgement of stents or catheters was reported in the EMR.

The primary outcome of the study was occurrence of UTIs within 90 days of surgery, defined as presence of all the following symptoms (fever and/or flank/abdominal pain and/or general malaise) or physician suspicion, urine culture with ≥ 10,000 colony-forming units (CFU), and antibiotic/antifungal treatment. All cases of UTIs, pyelonephritis, and urosepsis within 90 days of surgery were reported and included in the analysis.

Variables tested as risk factors for postoperative UTI were sex, age (grouped in quartiles), preoperative body mass index (*BMI*), diabetes, hypertension, CCI (grouped by 0–3 and ≥ 4), preoperative eGFR (grouped by CKD stages 1–2 and 3–5), type of urinary diversion, and early stent removal or dislodgement.

The renography scans were Tc-99 m-MAG3 renograms. They were routinely performed 6–8 weeks after surgery, but due to occasional variations including cases with prolonged stenting, the renography scans for included patients were performed from postoperative day 14 until postoperative day 308. As the results from renography scans are interpreted by the treating clinicians, and some degree of delayed excretion can be considered expected after surgery, delayed excretion was defined as any documentation thereof by a urologist in the EMR. Asymmetrical functional distribution was defined as relative renal uptake ratio of 40% or less in either kidney.

Baseline characteristics were analyzed using descriptive statistics; categorical variables were presented as percentages and frequencies and continuous variables were presented as medians and interquartile ranges (IQR). Time from operation to UTI was presented with cumulative incidence curves and cause-specific cox regression analysis was performed to assess the impact of potential risk factors on the time to first postoperative UTI. Death before event was defined as the competing risk. The cox regression analysis only included patients with ileal conduits, orthotopic neobladder, and continent cutaneous reservoirs. The association between findings on renography scans and occurrence of postoperative UTIs were investigated using multivariable logistic regression. Hazard ratios (HR) or odds ratios (OR) with 95% confidence intervals (CI) were calculated for each variable and the significance level was set to *p* < 0.05.

All statistical analyses were performed using R (version 2024.09.1 + 394).

## Results

A total of 612 patients were included in the study. Baseline characteristics are summarized in Table 1. The median age was 69 (IQR = 62–75) at the time of surgery, 32% (*N* = 198) were female, and 21% (*N* = 127) had CCI ≥ 4. Most patients (88%, *N* = 538) were operated due to bladder cancer, and 4.9% (*N* = 30) were operated due to other bladder-invading cancers, including prostate, vaginal/cervical, and intestinal tumors, while 7.2% (*N* = 44) were operated due to benign conditions, with the two most common being adverse effects of radiotherapy (*N* = 23) and painful bladder syndrome (*N* = 11).

**Table 1:** Patient characteristics

Characteristics	No or median	Percentage (%) or IQR
Total number of patients	612	100
Sex		
Female	198	32
Male	414	68
Age at time of surgery	69	62–75
Preoperative BMI	26	23–29
< 18.5 kg/m^2^	19	3.1
18.5–24.9 kg/m^2^	246	40
25–29.9 kg/m^2^	232	38
30–34.9 kg/m^2^	87	14
≥ 35 kg/m^2^	25	4.2
Smoking status		
Never	122	20
Previous smoker	306	50
Current smoker	181	30
Preoperative Charlson Comorbidity Index		
0- 3	485	79
4- 8	127	21
ASA-score		
1- 2	345	56
3	265	43
4	2	0.3
Preoperative hypertension	277	45
Preoperative diabetes	73	12
Preoperative eGFR (mL/min)	77	58–93
Preoperative CKD stage		
Stage 1 (eGFR ≥ 90 mL/min)	173	28
Stage 2 (eGFR 60 − 89 mL/min)	280	46
Stage 3a (eGFR 45 − 59 mL/min)	85	14
Stage 3b (eGFR 30 − 44 mL/min)	56	9.2
Stage 4 (eGFR 15 − 29 mL/min)	15	2.5
Stage 5 (eGFR < 15 mL/min)	3	0.5
Urinary diversion type		
Ileal conduit	546	89
Orthotopic neobladder	26	4.3
Continent cutaneous reservoir	28	4.6
Mitrofanoff procedure	6	1.0
Uretero-cutaneous ostomy	6	1.0
Indication for urinary diversion		
Urothelial cancer	538	88
NMIBC	233	44
MIBC	300	56
Other cancer	30	4.9
Cervical/vaginal	19	63
Prostate	4	12
Other	7	23
Benign	44	7.2
Adverse effects of radiotherapy	23	52
Painful bladder syndrome	11	25
Damage after surgery	3	6.8
Intractable incontinence	4	9.1
Other	3	6.8
History of radiotherapy to the pelvis	71	12
Preoperative chemotherapy	119	19
Open surgery	399	65
Robot-assisted laparoscopy	206	34
Converted from robot-assisted to open	7	1.1
Concomitant cystectomy	588	96
Postoperative stent removal		
Planned removal	555	91
Emergency removal due to complication	13	2.1
Dislodged spontaneously	42	6.9

Missing data: BMI = 3; smoking status = 3; preoperative T-stage = 4

*IQR* Interquartile range*, BMI* Body mass index*, ASA* American Society of Anaesthesiology*, NMIBC* Non-muscle invasive bladder cancer*, MIBC* Muscle invasive bladder cancer*, EGFR* Estimated glomerular filtration rate*, CKD* Chronic kidney disease

^*^2 patients did not have stents placed during surgery

Ileal conduit was the most common urinary diversion, which 89% (*N* = 546) received, while 4.3% (*N* = 26) received an orthotopic neobladder, 4.6% (N = 28) received a continent cutaneous reservoir, 1% (*N* = 6) received a Mitrofanoff procedure, and 1% (*N* = 6) received a ureterocutaneostomy. In 9% (*N* = 55) of cases, the stents or catheters were either removed earlier than planned or spontaneously dislodged. 

A total of 346 episodes of UTI were identified in 242 patients (40%) within 90 days after surgery (Table 2), and 79 patients (13% of the total population) experienced two or more UTIs. Of the patients who received an ileal conduit, 36% (N=198) experienced at least one UTI, while 58% (N=15) of the patients with an orthotopic neobladder and 75% (N=21) of the patients with a continent cutaneous reservoir had a least one UTI. Of the 346 UTIs, bacteremia occurred in 32% (N=111), and 70% UTI episodes (N=243) caused a hospital readmission, while 23% (N=78) occurred during primary or subsequent admissions.

**Table 2:** Postoperative UTIs

	Number	Percentage
Number of patients with postoperative UTI	242	40
One instance		
Total population (*n* = 612)	163	27
Ileal conduit (*n* = 546)	142	26
Orthotopic neobladder (*n* = 26)	9	35
Continent cutaneous reservoir (*n* = 28)	7	25
Mitrofanoff procedure (*n* = 6)	2	33
Ureterocutaneostomy (*n* = 6)	3	50
Two or more instances		
Total population (*n* = 612)	79	13
Ileal conduit (*n* = 546)	56	10
Orthotopic neobladder (*n* = 26)	6	23
Continent cutaneous reservoir (*n* = 28)	14	50
Mitrofanoff procedure (*n* = 6)	2	33
Ureterocutaneostomy (*n* = 6)	1	17
Total number of UTIs	346	100
Fever	323	93
Bacteremia	111	32
Hydronephrosis	120	35
Hospitalization		
Already under admission at UTI onset	78	23
UTI causing hospital admission	243	70
Out-patient management of UTI	25	7.2

**Fig. 1:**
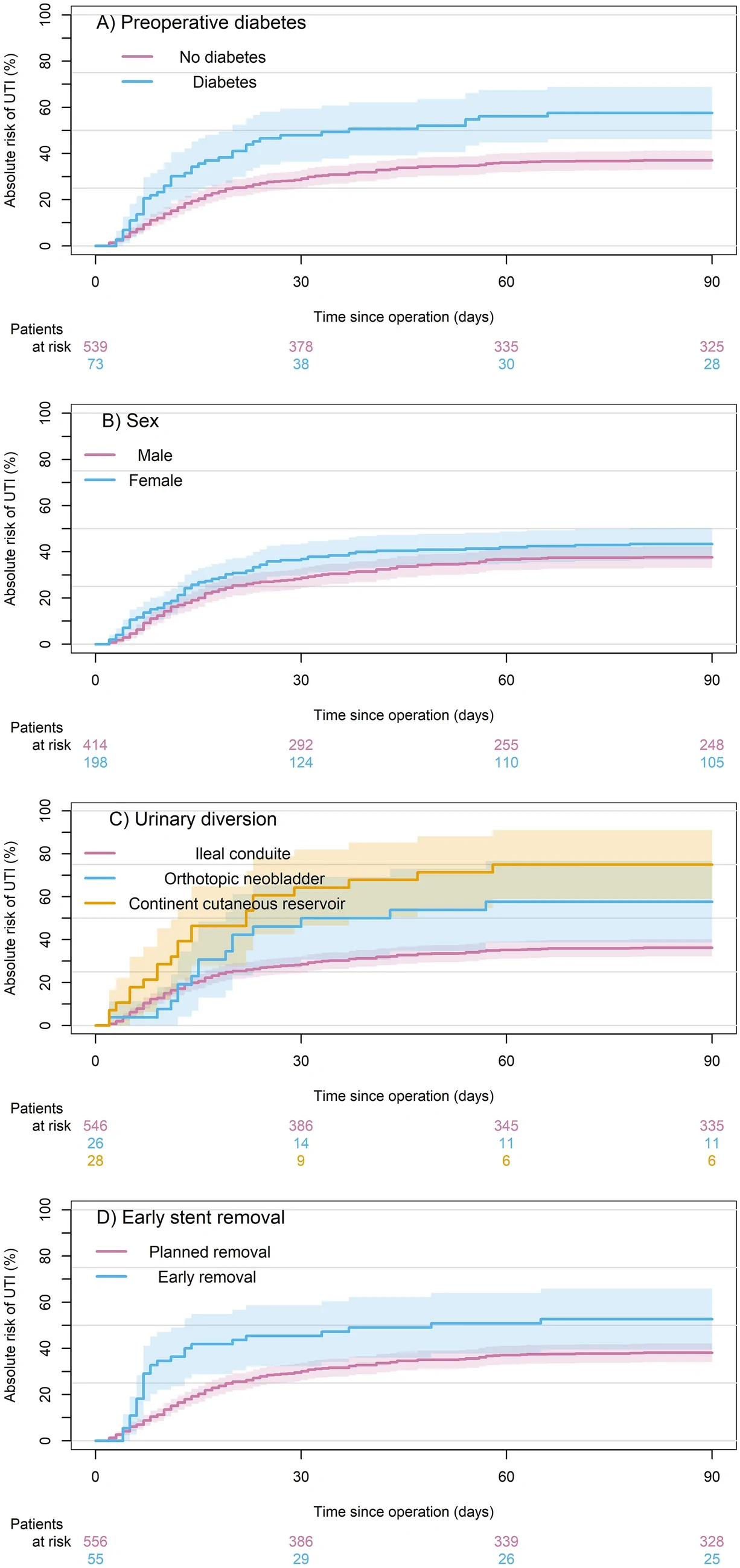
Cumulative incidence curves of the risk of getting a UTI stratified for **A** preoperative diabetes, **B** sex, **C** type of urinary diversion, and **D** early stent removal/dislodgement

Figure 1 presents cumulative incidence curves of the risk of developing a postoperative UTI, stratified by sex, preoperative diabetes, type of urinary diversion, and early removal or dislodgement of stents (Fig. 1).

 A cause-specific Cox-regression analysis of the association between developing at least one UTI and possible risk factors is shown in Supplementary material 3. Female sex (HR=1.5, 95%CI=1.2-2.1), BMI 25-30 kg/m^2^ (HR=1.6, 95%CI=1.2-2.2), preoperative diabetes (HR=1.7, 95%CI=1.1-2.5), orthotopic neobladder (HR=2.1, 95%CI=1.2-3.7) or continent cutaneous reservoir (HR=3.4, 95%CI=2.1-5.6) as diversion type compared to ileal conduit, and early removal or dislodgement of ureteral stents (HR=2.0, 95%CI=1.4-3.1) were all independent risk factors of UTI after surgery.

 Of all the patients, 85% (N=519) had a renography scan around 6-8 weeks after surgery (me-dian time=52 days) as part of their follow-up program, and 58% (N = 290) of the scans were assessed to have delayed excretion, while 23% (N=120) showed asymmetrical functional dis-tribution of the kidneys. A repeated renography scan was performed in 3.8% (N=11) of pa-tients with delayed excretion and in 6.7% (N=8) of patients with asymmetrical functional dis-tribution. Meanwhile, 9.0% (N=26) of patients with delayed excretion and 11% (N=13) of those with asymmetrical functional distribution got a stent to relieve the kidney. More than 80% of abnormal renography results did not lead to subsequent stenting nor repeat scan. Supplementary material 4 shows a table summarizing the characteristics of the postoperative renography scans.

 Supplementary material 3 also presents a multivariable logistic regression analysis showing that having a postoperative UTI was an independent risk factor of delayed excretion (OR=1.7, 95%CI=1.1-2.6) but was not associated with asymmetrical functional distribution of the kidneys. 

## Discussion

This study identified a high rate of postoperative UTIs in patients who have undergone urinary diversion, with 40% of the patients experiencing at least one episode of UTI within 90 days after surgery. Many of the UTIs were complicated, involving fevers and bacteremia, and a large proportion required readmission to the hospital, showing that the patient group is prone to severe UTIs, often progressing to urosepsis requiring more intensive treatment and longer hospital stays. More than half of the patients had abnormal postoperative renography scans, which were also related to postoperative UTIs.

Incidence rates of postoperative UTI have previously been reported for cystectomy cohorts to be around 10–40% [4–9, 13–15]. The rate in the current study is in the higher end of the rates reported in the literature, and there are several possible explanations for this. First, the defined postoperative period varies between studies, so reported rates include some studies investigating 30-day rates and some investigating 90-day rates [4–10, 13, 14]. Second, at our center, patients are instructed to contact the department directly at any sign of infection, and all contacts are registered with clinical notes in the national EMR, which substantially minimizes the risk of missing data from private clinics or general practitioners. Third, this study defined a significant urine culture to be ≥ 10,000 CFU, while other studies used ≥ 100,000 CFU [4, 7, 8, 16]. Although the threshold of 10,000 CFU was deliberately chosen as a pragmatic threshold for this patient group that is known to be more susceptible to UTIs, the difference in definitions may lead to an overestimation of UTIs in the study compared to other studies.

Existing literature lacks consensus on risk factors for developing a UTI after urinary diversion. While one study has found female sex and hypertension as risk factors, other studies have identified higher CCI, preoperative diabetes, and perioperative blood transfusion as risk factors [5, 6, 8]. Additional factors, such as the use of intraoperatively placed ureteral stents and choice and duration of antibiotic prophylaxis, have also been considered as potential risk factors for postoperative infections [3]. Finally, continent urinary diversions, including orthotopic neobladder, have repeatedly been reported as a significant and independent risk factor for development of postoperative UTIs [7, 13, 15, 17]. This may be due to intermittent self-catheterization, incomplete voiding with residual urine volumes, which creates an optimal environment for bacteria and extended use of ureteral stents postoperatively [18].

Among the risk factors identified in this analysis, we found that patients with early stent dislodgement were more than twice as likely to experience a UTI as patients with planned removal. This confirms our previously published data, showing that there is a clear peak in UTIs around stent removal [10, 14]. It also indicates that the antimicrobial prophylaxis administered during planned removal could play a significant role in reducing infections, but that the prophylactic strategy can be improved [14, 19]. A retrospective study has found that the use of intraoperative ureteral stents for ileal conduits when compared with procedures without the use of stents was associated with a greater risk of infectious complications, suggesting that a stentless procedure could be considered.[20].

Patients with a postoperative UTI had a nearly twofold risk of delayed excretion on renography scans. This association indicates that a major reason for developing UTI after construction of a diversion is obstruction of the urinary tract. Conversely, a UTI may contribute to obstruction problems. While the directionality remains unclear, clinicians should consider the association between UTI and urinary tract obstruction during management of patients with a urinary diversion. Nonetheless, the data also indicate that in a population with systematic use of postoperative renography scans, the vast majority of renography scans showing an abnormal result do not lead to clinical action. This raises the question of whether routine use of renography scans can be omitted as a standard of care in the follow-up program, and rather be offered to a defined group of patients with postoperative UTIs or renal function decline.

This is, to our knowledge, the first study investigating the role of routinely performed renography scans after construction of urinary diversions. The study provides valuable knowledge at a period when global healthcare resources are scarce. Furthermore, most studies within this field only include bladder cancer or cystectomy patients, but we expanded the patient cohort to include all patients with any type of a urinary diversion based on any indication, thereby including a patient group that has historically been understudied. Another strength lays in the high granularity of the Danish EMR. Missing data are expected to be minimal in this patient group that is almost entirely treated at the public hospitals.

The findings could be supported by long-term outcomes, such as long-term renal function, incidence of ureteral strictures, and chronic recurrent UTIs. As the current study investigated short-term follow-up of this population, long-term data were not available. Further, the threshold for abnormal findings on renography scans contains some degree of interpretation introducing a degree of subjectivity. The relationship between the extend of asymmetrical distribution or delayed excretion and the need for and success of intervention should be further investigated, preferably in a setting with a higher level of inter-rater reliability. Finally, the rarer types of urinary diversions lack larger population sizes for high-quality data analysis. Fewer than 10% of the patients received a continent diversion, limiting statistical power and generalizability.

The data were collected from a single center; therefore, the generalizability of the results may be uncertain, as institutional practices and patient demographics may differ elsewhere.

## Conclusions

This study confirms a high incidence of postoperative UTI after construction of a urinary diversion, with a substantial proportion complicated by bacteremia. Several risk factors associated with the development of UTIs following urinary diversion were identified, the most dominant being continent urinary diversion types and early removal or dislodgement of the ureteral stents from a center that routinely performs postoperative renography scans around 6–8 weeks after surgery, we found that urologist-evaluated delayed excretion was linked with postoperative UTIs. Only a minor proportion of abnormal renography results led to subsequent intervention, indicating the potential for a more selective and resource-conserving use of renography scans in the postoperative course of urinary diversion procedures.

## Supplementary Information

Below is the link to the electronic supplementary material.

Supplementary file1 (PDF 459 KB)

## Data Availability

The data that support the findings of this study are not openly available due to reasons of sensitivity but can be made available upon reasonable request from the authors. Data are safely stored in a controlled server at Capital Region of Denmark.

## References

[1] DaBlaCa. Dansk blaere cancer database (DaBlaCa-Data) Årsrapport 2024 [Internet]. 2024. www.sundk.dk

[2] Maibom SL, Joensen UN, Poulsen AM, Kehlet H, Brasso K, Røder MA, BMJ Publishing Group (2021) Short-term morbidity and mortality following radical cystectomy: a systematic rev. BMJ Open. 10.1136/bmjopen-2020-043266 33853799 10.1136/bmjopen-2020-043266 PMC8054090

[3] Antonelli L, Sebro K, Lahmar A, Black PC, Ghodoussipour S, Hamilton-Reeves JM et al (2023) Association between antibiotic prophylaxis before cystectomy or stent removal and infection complications: a systematic review. Eur Urol Focus. 10.1016/j.euf.2023.01.012 36710211 10.1016/j.euf.2023.01.012 PMC13071900

[4] Clifford TG, Katebian B, Van Horn CM, Bazargani ST, Cai J, Miranda G et al (2018) Urinary tract infections following radical cystectomy and urinary diversion: a rev of 1133 patients. World J Urol 36:775–781. 10.1007/s00345-018-2181-2 29372354 10.1007/s00345-018-2181-2

[5] Sandberg M, Vancavage R, Refugia JM, Underwood G, Ye E, Marie-Costa C et al (2024) Post-operative urinary tract infections after radical cystectomy: incidence, pathogens, and risk factors. J Clin Med. 10.3390/jcm13226796 39597940 10.3390/jcm13226796 PMC11594803

[6] Parker WP, Toussi A, Tollefson MK, Frank I, Thompson RH, Zaid HB et al (2016) Risk factors and microbial distribution of urinary tract infections following radical cystectomy. Urology 94:96–101. 10.1016/j.urology.2016.03.049 27125878 10.1016/j.urology.2016.03.049

[7] Ghoreifi A, Van Horn CM, Xu W, Cai J, Miranda G, Bhanvadia S et al (2020) Urinary tract infections following radical cystectomy with enhanced recovery protocol: a prospective study. Urol Oncol Semin Orig Investig 38:75.e9-75.e14. 10.1016/j.urolonc.2019.12.021 10.1016/j.urolonc.2019.12.021 31956079

[8] Lu X, Jiang H, Wang D, Wang Y, Chen Q, Chen S et al (2022) Early warning models to predict the 90-day urinary tract infection risk after radical cystectomy and urinary diversion for patients with bladder cancer. Front Surg. 10.3389/fsurg.2021.782029 35127802 10.3389/fsurg.2021.782029 PMC8814316

[9] Kim KH, Yoon HS, Yoon H, Chung WS, Sim BS, Lee DH (2016) Febrile urinary tract infection after radical cystectomy and ileal neobladder in patients with bladder cancer. J Korean Med Sci 31:1100–1104. 10.3346/jkms.2016.31.7.1100 27366009 10.3346/jkms.2016.31.7.1100 PMC4901003

[10] Vejlgaard M, Maibom SL, Joensen UN, Moser C, Røder A (2024) Microbial trends in infection-related readmissions following radical cystectomy for bladder cancer. Urology 183:134–140. 10.1016/j.urology.2023.09.007 37742848 10.1016/j.urology.2023.09.007

[11] Inker LA, Eneanya ND, Coresh J, Tighiouart H, Wang D, Sang Y et al (2021) New creatinine- and cystatin C-based equations to estimate GFR without race. N Engl J Med 385:1737–1749. 10.1056/nejmoa2102953 34554658 10.1056/NEJMoa2102953 PMC8822996

[12] Dablaca. Klinisk Retningslinje │Kraeft [Internet]. 2025. https://www.dmcg.dk/kliniske-retningslinjer/kliniske-retningslinjer-opdelt-paa-dmcg/cancer-i-urinvejene/blaerekraeft/f.

[13] Aldhaam NA, Hussein AA, Elsayed AS, Jing Z, Osei J, Kurbiel Z et al (2021) Detailed analysis of urinary tract infections after robot-assisted radical cystectomy. J Endourol 35:62–70. 10.1089/end.2020.0316 32664741 10.1089/end.2020.0316

[14] Kolwijck E, Seegers AEM, Tops SCM, Van Der Heijden AG, Sedelaar JPM, Ten Oever J (2019) Incidence and microbiology of post-operative infections after radical cystectomy and ureteral stent removal; a retrospective cohort study. BMC Infect Dis. 10.1186/s12879-019-3932-4 30943902 10.1186/s12879-019-3932-4 PMC6448312

[15] Mano R, Goldberg H, Stabholz Y, Hazan D, Margel D, Kedar D et al (2018) Urinary tract infections after urinary diversion different occurrence patterns in patients with ileal conduit and orthotopic neobladder. Urology 116:87–92. 10.1016/j.urology.2018.03.042 29626568 10.1016/j.urology.2018.03.042

[16] Haider M, Ladurner C, Mayr R, Tandogdu Z, Fritsche HM, Fradet V et al (2019) Use and duration of antibiotic prophylaxis and the rate of urinary tract infection after radical cystectomy for bladder cancer: results of a multicentric series. Urol Oncol Semin Orig Investig 37:300.e9-300.e15. 10.1016/j.urolonc.2019.01.017 10.1016/j.urolonc.2019.01.017 30871997

[17] Nazmy M, Yuh B, Kawachi M, Lau CS, Linehan J, Ruel NH et al (2014) Early and late complications of robot-assisted radical cystectomy: a standardized analysis by urinary diversion type. J Urol 191:681–687. 10.1016/j.juro.2013.10.022 24099746 10.1016/j.juro.2013.10.022

[18] Mirto BF, Barone B, Balsamo R, Abate M, Caputo VF, Sciarra A et al (2024) Early and late post-procedural complications in different orthotopic neobladder surgical approaches: a systematic rev. Surg Oncol 55:102090. 10.1016/j.suronc.2024.102090 38917777 10.1016/j.suronc.2024.102090

[19] Vejlgaard M, Stroomberg HV, Ynddal MS, Moser C, Joensen UN, Røder A (2025) Multicentre, open-label, phase IV, randomised trial testing superiority of individualised targeted antibiotic prophylaxis over empiric prophylaxis at ureteral stent removal following cystectomy: study protocol for the REINFORCE trial. Trials. 10.1186/s13063-025-08981-w 40722189 10.1186/s13063-025-08981-w PMC12302692

[20] Donat SM, Tan KS, Jibara G, Dalbagni G, Carlon VA, Sandhu J (2021) Intraoperative ureteral stent use at radical cystectomy is associated with higher 30-day complication rates. J Urol 205:483–490. 10.1097/JU.0000000000001329 33238829 10.1097/JU.0000000000001329 PMC8162033

