# Supplementary material for: Postoperative urinary tract infections and the role of Tc-99 m-MAG3 renography scans following construction of a urinary diversion
Source: Int Urol Nephrol. 2026 Mar 17;58(9):3483–90. doi: 10.1007/s11255-026-05049-9 (PMC13506627; doi:10.1007/s11255-026-05049-9)
Supplement: Supplementary file 1 [file 11255_2026_5049_MOESM1_ESM.pdf]

Supplementary material

Article title: Postoperative urinary tract infections and the role of Tc-99m-MAG3 renography scans following construction of a urinary diversion

Journal name: International Urology and Nephrology

Authors: Julie Sofie Riemann[a], Ulla Nordström Joensen[a], Andreas Røder[a], Maja Vejlgaard[a]

[a] Department of Urology, Centre for Cancer and Organ Diseases, Copenhagen University Hospital – Rigshospitalet, Copenhagen Denmark; Department of Clinical Medicine, Faculty of Health and Medical Sciences, University of Copenhagen, Copenhagen, Denmark

Corresponding author: Julie Sofie Riemann

E-mail: Julie.sofie.riemann.02@regionh.dk

Supplementary material 1: Operational procedures

Construction of an ileal conduit:

Approximately 15 cm of the distal ileum is mobilized approximately 20-30 cm from the ileocecal valve to form the conduit. The intestinal continuity is reestablished by hand-sewn or stapled side-to-side anastomosis. Both ureters are anastomosed end-to-side to the proximal end of the conduit and the distal end is passed through the abdominal wall to form a urostomy. Baby feeding tubes are placed in both ureters and sutured to the peristomal skin. The tubes are removed approximately ten days postoperatively.

Construction of an orthotopic neobladder:

Approximately 50 cm of the distal ileum is mobilized approximately 20-30 cm from the ileocecal valve. The intestinal continuity is reestablished by hand-sewn or stapled side-to-side anastomosis. The isolated ileal segment is detubularized and folded to form the neobladder, anastomosed to the urethra, and the ureters are anastomosed end-to-side

to the neobladder. Prior to closing the neobladder, double-J stents are placed in both ureters and secured to a urethral catheter to ensure postoperative drainage. The catheters with attached double-J stents are removed approximately three weeks postoperatively.

Construction of a continent cutaneous reservoir:

The continent cutaneous urinary diversion performed at our center is the Indiana pouch. The ascending colon and approximately 10-15 cm of distal ileum is mobilized to form the reservoir. The ileocolonic continuity is reestablished by hand-sewn side-to-side anastomosis. The isolated colonic segment is detubularized and hand-sewn into a sphere to form the reservoir. The ureters are anastomosed to the posterior aspect of the reservoir over double-J stents. The ileal limb is narrowed, and a catheter is passed through and secured to the double-J stents before closing the reservoir. The ileal catheterization channel is passed through the abdominal wall to form a flush stoma. The catheter with attached double-J stents is removed approximately three weeks postoperatively.

Mitrofanoff procedure:

The appendix is mobilized with its mesentery and removed from the cecum. The distal end of the appendix is opened and anastomosed to the bladder, and the base of the appendix is passed through the abdominal wall to form a catheterization channel. A catheter is placed in the canal. The catheter is removed approximately three weeks postoperatively.

Construction of ureterocutaneostomies:

The distal ends of the ureter(s) are passed through the abdominal wall to form a flush stoma and stents are placed. The ureteral stents are usually replaced every 8-12 weeks postoperatively in the out-patient clinic.

Supplementary material 2: Illustration of follow-up after cystectomy and urinary diversion.

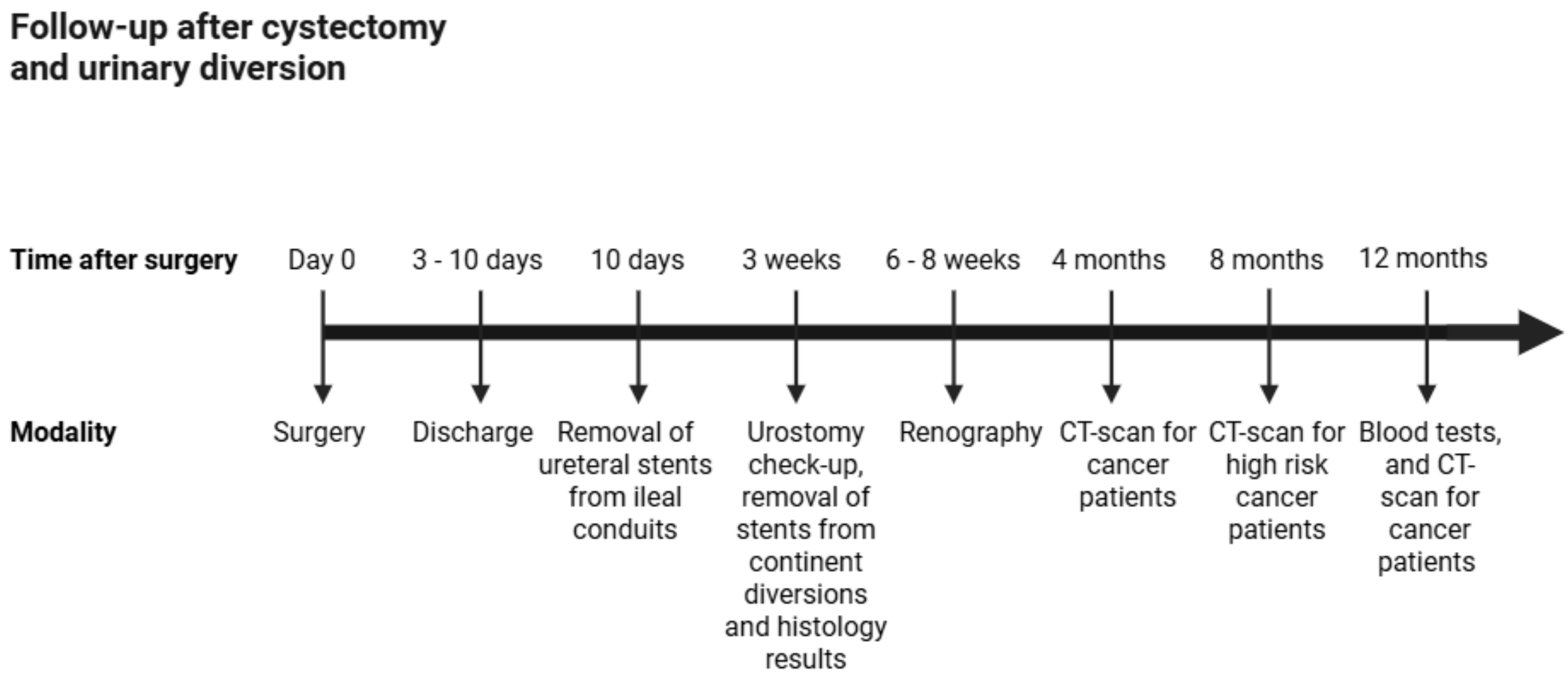

Illustration of timeline of the standard-of-care follow-up program after cystectomy and urinary diversion at Rigshospitalet, Copenhagen, Denmark

Supplementary material 3: Risk factor analysis.

Cause-specific cox regression analysis of having at least one UTI

	Hazard ratio	95%CI	P-value
Female sex	1.5	(1.1-2.0)	0.0044
Age (years)			
< 62.1	Ref	Ref	Ref
62.1 - 69.1	0.77	(0.53-1.1)	0.17
69.1 – 74.9	0.93	(0.63-1.4)	0.73
> 74.9	0.84	(0.56-1.3)	0.42
BMI			
20-25 kg/m2	Ref	Ref	Ref
<20 kg/m2	1.3	(0.58-2.8)	0.56
25-30 kg/m2	1.6	(1.1-2.1)	0.0047
>30 kg/m2	1.4	(0.93-2.0)	0.12
Preoperative diabetes	1.7	(1.2-2.6)	0.0066
Preoperative hypertension	1.2	(0.93-1.7)	0.14
Diversion type			
Ileal conduit	Ref	Ref	Ref
Orthotopic neobladder	2.1	(1.2-3.6)	0.0090
Continent cutaneous reservoir	3.4	(2.1-5.6)	< 0.001
Charlson Comorbidity Index ≥ 4	1.1	(0.79-1.6)	0.48
Preoperative CKI stage			
Stage 1-2	Ref	Ref	Ref
Stage 3a-5	1.1	(0.81-1.5)	0.55
Early stent removal or dislodgement	2.0	(1.3-3.0)	< 0.001

Multivariable logistic regression of abnormal renography results

	Delayed excretion			Asymmetrical functional distribution		
	Odds Ratio	95%CI	p-value	Odds Ratio	95%CI	p-value
Female sex	0.66	(0.43-1.0)	0.06	0.97	(0.59-1.6)	0.90
Age (years)						
< 62	Ref	Ref	Ref	Ref	Ref	Ref
62 - 69	1.1	(0.64-1.9)	0.72	1.1	(0.58-2.2)	0.72
69 - 75	1.8	(0.99-3.1)	0.053	0.92	(0.46-1.8)	0.81
> 75	1.9	(1.0-3.4)	0.047	1.1	(0.52-2.1)	0.88
Preoperative diabetes	1.3	(0.84-3.5)	0.14	1.0	(0.49-2.2)	0.92
Preoperative CKI stage > 2	3.7	(2.3-6.7)	< 0.001	4.2	(2.6-6.8)	< 0.001
Postoperative UTI	1.6	(1.0-2.4)	0.034	0.97	(0.61-1.5)	0.88
Type of urinary diversion						
Ileal conduit	Ref	Ref	Ref	Ref	Ref	Ref
Orthotopic neobladder	1.4	(0.55-3.5)	0.48	0.71	(0.20-2.6)	0.60
Continent cutaneous reservoir	0.93	(0.37-2.3)	0.87	0.87	(0.27-2.8)	0.82

Supplementary material 4:

Overview of renography scan characteristics and intervention after abnormal findings

	Number	Percentage (%) or IQR
Total number of patients with at least one post-operative renography	519	85
Median time from operation to first renography (days)	52	48 - 59
Median eGFR at the time of first renography	74	55 - 91
**Delayed excretion at first postoperative renography (reported by treating clinician)**	**290**	**58**
Intervention:		
No follow-up	253	87
Follow-up renography only	11	3.8
Postoperative stenting	26	9.0
**Asymmetrical functional distribution**	**120**	**23**
Intervention:		
No follow-up	99	83
Follow-up renography only	8	6.7
Postoperative stenting	13	11

IQR = interquartile range
